# Deciphering metabolic crosstalk in context: lessons from inflammatory diseases

**DOI:** 10.1002/1878-0261.13588

**Published:** 2024-01-26

**Authors:** Fenne W. M. Verheijen, Thi N. M. Tran, Jung‐Chin Chang, Femke Broere, Esther A. Zaal, Celia R. Berkers

**Affiliations:** ^1^ Division of Cell Biology, Metabolism & Cancer, Department Biomolecular Health Sciences, Faculty of Veterinary Medicine Utrecht University The Netherlands; ^2^ Division of Infectious Diseases and Immunology, Department Biomolecular Health Sciences, Faculty of Veterinary Medicine Utrecht University The Netherlands; ^3^ Biomolecular Mass Spectrometry and Proteomics, Bijvoet Centre for Biomolecular Research Utrecht University The Netherlands

**Keywords:** advanced metabolomics methods, immunometabolism, inflammatory diseases, metabolic crosstalk, metabolic heterogeneity, microenvironment

## Abstract

Metabolism plays a crucial role in regulating the function of immune cells in both health and disease, with altered metabolism contributing to the pathogenesis of cancer and many inflammatory diseases. The local microenvironment has a profound impact on the metabolism of immune cells. Therefore, immunological and metabolic heterogeneity as well as the spatial organization of cells in tissues should be taken into account when studying immunometabolism. Here, we highlight challenges of investigating metabolic communication. Additionally, we review the capabilities and limitations of current technologies for studying metabolism in inflamed microenvironments, including single‐cell omics techniques, flow cytometry‐based methods (Met‐Flow, single‐cell energetic metabolism by profiling translation inhibition (SCENITH)), cytometry by time of flight (CyTOF), cellular indexing of transcriptomes and epitopes by sequencing (CITE‐Seq), and mass spectrometry imaging. Considering the importance of metabolism in regulating immune cells in diseased states, we also discuss the applications of metabolomics in clinical research, as well as some hurdles to overcome to implement these techniques in standard clinical practice. Finally, we provide a flowchart to assist scientists in designing effective strategies to unravel immunometabolism in disease‐relevant contexts.

## Graphical abstract

The local microenvironment has a profound impact on the metabolism of immune cells. Therefore, immunological and metabolic heterogeneity as well as the spatial organization should be considered when studying immunometabolism. In this review, we discuss important challenges in immunometabolism research, highlight the state‐of‐the‐art techniques suited to address these challenges, and indicate advancements that can push the field forward.

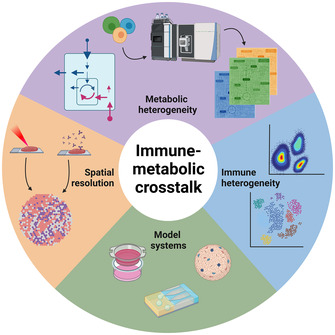

**Abbreviations**
CITE‐seq: cellular indexing of transcriptomes and epitopes by sequencing
CyTOF: cytometry by time of flight
DESI: desorption electrospray ionization
GC: gas chromatography
LC: liquid chromatography
MALDI: matrix‐assisted laser desorption/ionization
MS: mass spectrometry
MSI: mass spectrometry imaging
RA: rheumatoid arthritis
SCENITH: single‐cell energetic metabolism by profiling translation inhibition
SCM: single‐cell metabolomics
scMEP: single‐cell metabolomic regulome profiling
SIMS: secondary ion mass spectrometry
Tconvs: conventional T cells
Tregs: regulatory T cells

## 1 Introduction

### 1.1 Impact of immunometabolic interactions

Immune cell activation is accompanied by changes in metabolism. For example, naive T cells rely on oxidative phosphorylation and undergo a metabolic switch toward aerobic glycolysis upon activation [1, 2, 3]. Moreover, the metabolic states of immune cells can also regulate the differentiation and function of immune cells [4, 5, 6, 7]. For instance, tryptophan depletion, often occurring within the tumor microenvironment (TME), has been shown to induce a regulatory phenotype in T cells [8, 9]. These examples demonstrate the intricate yet tightly connected relationship between the phenotypes and metabolism of immune cells in normal and diseased states.

The metabolic and functional states of immune cells are profoundly influenced by the local environment [10, 11, 12, 13, 14, 15]. Both resident and infiltrating immune cells are influenced by tissue‐specific signals in the microenvironment, such as locally produced metabolites, growth factors, adhesion molecules, nutrients, and oxygen availability [16]. Immune cells also show a high degree of metabolic plasticity and express tissue‐specific transcription factors to adapt their nutrient utilization strategies in response to the local environment [11, 14, 17, 18]. For example, the differentiation of alveolar macrophages within the lipid‐enriched and oxygenated microenvironment of pulmonary alveoli is dependent on peroxisome proliferator‐activated receptor gamma—a transcription factor regulating lipid uptake and oxidation [19]. As a result, differentiated alveolar macrophages express a high level of CD36 to support the uptake of long‐chain fatty acids in the alveolar space and reduce fatty acid synthesis [20]. Similarly, bone‐marrow‐derived monocytes stepwise acquire the Kupffer cell identity in the liver driven by factors secreted by hepatocytes and liver sinusoidal endothelial cells, such as desmosterol and transforming growth factor‐beta (TGFβ) [21]. In turn, differentiated Kupffer cells upregulate metabolic programs that facilitate fatty acid synthesis and glycolysis in the nutrient‐enriched microenvironment of the liver [21, 22, 23].

Metabolic crosstalk between cells in the microenvironment further modulates inflammatory responses, for example, via metabolite signaling, cross‐feeding, or nutrient competition. Nutrient competition between cancer cells and immune cells in the TME is well known to contribute to an immune‐suppressive environment [5, 24, 25, 26, 27, 28]. In contrast, microenvironments in autoimmune diseases often drive an overactivation of immune cells [29, 30, 31, 32, 33]. Several metabolites, such as itaconate, lactate, and succinate, are now established as mediators of such (anti‐)inflammatory responses [34, 35, 36, 37]. For instance, itaconate derived from tumor‐associated macrophages or myeloid‐derived suppressor cells is taken up by infiltrating CD8^+^ cytotoxic T cells, leading to reduction in amino acid precursors for *de novo* nucleotide synthesis, proliferation, cytokine secretion, and cytolytic activity [38, 39]. Similar anti‐inflammatory effects of itaconate have been observed in an autoimmunity setting, where itaconate altered Th17/Treg differentiation via metabolic rewiring and epigenetic modulations toward a Treg lineage [40]. Lactate accumulation in the TME can suppress the proliferation and interferon‐γ secretion of local CD8^+^ cytotoxic T cells [41]. In contrast, lactate that builds up at inflamed sites can exacerbate the inflammatory response of CD4^+^ T cells [42]. Here, increased lactate uptake in CD4^+^ T cells led to enhanced IL‐17 production and elevated fatty acid synthesis. Consistently, inhibiting lactate transporter SLC5A12 ameliorates disease severity in a murine model of rheumatoid arthritis (RA) [42]. This central role of metabolism in regulating immune cell functionality and in inducing either inflammatory or immunosuppressive microenvironments makes cellular metabolism an interesting target to boost or dampen the immune response for the treatment of cancer or autoimmune diseases, respectively.

### 1.2 Challenges in studying immunometabolic interactions

**Fig. 1:**
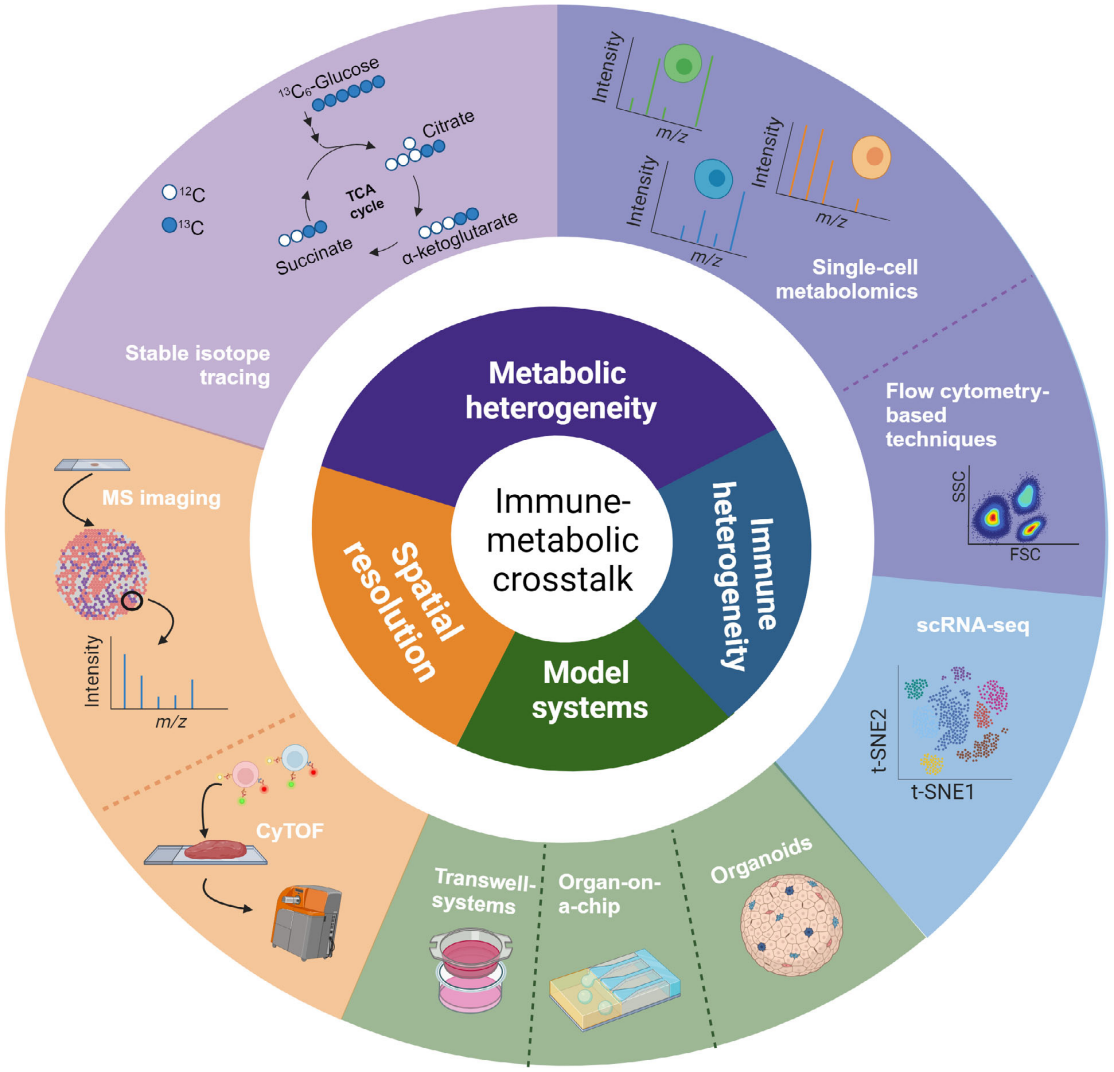
State‐of‐the‐art techniques to unravel immune and metabolic heterogeneity as well as spatial resolution in the context of inflammatory microenvironments. Several challenges prevent us from obtaining a clear view of the complex interplay of (immune) cells within physiological conditions. Within one sample, heterogeneity can occur at immune level, metabolic level, or a combination of the two. Several techniques can be utilized to unravel these levels of heterogeneity, such as single‐cell metabolomics (SCM), single‐cell RNA sequencing (scRNA‐seq), stable isotope tracing, and flow cytometry‐based techniques (Met‐flow and single‐cell energetic metabolism by profiling translation inhibition (SCENITH)). Moreover, to acquire more information on a spatial level, mass spectrometry imaging (MSI), such as matrix‐assisted laser desorption/ionization (MALDI‐TOF), or cytometry by time of flight (CyTOF) can be utilized to measure metabolites or metabolic proteins, respectively. Finally, sophisticated model systems, such as Transwell systems, organ‐on‐a‐chip, and organoids, can increase our knowledge of the impact of immunometabolism *in vivo*, as they more effectively mimic physiological conditions. Created with Biorender.com.

Studies in immunometabolism have revealed therapeutic targets and successful applications of metabolic drugs like rapamycin, mycophenolate, and methotrexate in controlling disease progression of rheumatoid arthritis (RA), systemic lupus erythematosus (SLE), and graft‐versus‐host diseases [40, 41, 42, 43, 44, 45, 46, 47]. However, several challenges remain in advancing our understanding of immunometabolism and immunometabolic crosstalk (Fig. 1). Overcoming these challenges is crucial for identifying mechanism‐based immunometabolic targets and harnessing immunometabolism for the development of novel therapeutic strategies.

Much of our current knowledge of immunometabolism in health and disease is based on bulk metabolomics and/or other bulk metabolic analyses in different immune cell types [43, 44, 45, 46, 47]. As these analyses do not provide information on metabolic heterogeneity in the microenvironment, they overlook metabolic features of minor subsets that are potentially important for disease pathogenesis [48]. Advances in single‐cell RNA sequencing (scRNA‐seq) have already led to in‐depth characterization of the immune landscape in healthy and diseased tissues at the single‐cell level [49, 50, 51, 52]. Although scRNA‐seq is efficient in charting heterogeneous metabolic phenotypes, it does not provide information on actual metabolite levels, making the study of metabolic crosstalk challenging. Integration of metabolic heterogeneity and immune heterogeneity at the single‐cell level will greatly advance our understanding of metabolism in disease pathogenesis. This will ultimately enable the identification of effective metabolic targets in the key subsets of immune cells and facilitate the design of effective drug delivery strategies while minimizing systemic toxicities [53, 54, 55, 56].

In addition, because the cellular organization of tissues determines if and how cells can engage in metabolic crosstalk, spatial resolution of cell distribution and their metabolic profiles is crucial for understanding metabolic interactions in tissue contexts. Commonly used techniques for studying metabolism often require cell isolation, thereby losing information about cellular structure within a tissue. Moreover, metabolites themselves are often distributed within a tissue, and linking metabolite distributions to spatial distribution of cell types can boost our knowledge of metabolic interactions that can aid in improving healthcare. For example, spatial (immune) metabolic information differentiating healthy from diseased tissue can enhance secure removal of diseased tissue during surgery [57]. Identifying immune metabolic targets expressed on diseased tissue but not on the surrounding healthy tissue can enable us to develop drugs that specifically target the diseased sections within a tissue, increasing specificity and reducing toxicity [58, 59].

Finally, there is an urgent need for *in vitro* models that better represent the tissue contexts as metabolic adaptation by immune cells varies between *in vitro* and *in vivo* systems. For example, *in vitro* bioenergetic profiling of CD8^+^ T cells isolated from *Listeria‐*infected mice revealed hallmarks of aerobic glycolysis; in contrast, the *in vivo* counterparts exhibit higher rates of oxidative metabolism and diverging use of downstream metabolite pyruvate [60]. The most widely used *in vitro* models in immunometabolism research [61, 62, 63, 64] include myeloid cells derived from peripheral blood mononuclear cells and bone marrow as well as lymphocytes derived from lymph nodes and the spleen [61, 62, 63, 64]. However, *in vitro* conditions often do not correctly represent *in vivo* conditions. For example, serum, which provides environmental signals within the circulating blood, is often added to *in vitro* cultured cells but may not contain the tissue‐specific signals that resident and infiltrating immune cells encounter. Likewise, nutrient levels in most cell‐culture media do not match nutrient levels within the immune microenvironments [9]. In addition, most cell culture models do not allow for the study of metabolic crosstalk between cells in the immune microenvironment. The development of complex *in vitro* models that can be interrogated for their metabolic status and allow mapping of metabolic crosstalk would greatly advance our understanding of physiological metabolic adaptation to local and disease‐active microenvironments by different cell types.

In this review, we discuss the state‐of‐the‐art methods that are currently used in immunometabolism research, including single‐cell technologies, flow cytometry‐based metabolic profiling techniques, imaging methods, and advanced co‐culture systems. We describe the advantages and limitations of each of these techniques and exemplify how each can be used in immunometabolism studies to address one or more of the challenges described above. We also consider how advanced metabolomics methods can contribute to diagnostics and precision medicine in future clinical practice, providing guidance in choosing methods for specific immunometabolic research questions.

## 2 Technical advances in immunometabolism research

### 2.1 Exploring immune and metabolic heterogeneity on the single‐cell level

#### 2.1.1 Single‐cell omics methods for mapping immune and metabolic heterogeneity: scRNA‐seq and single‐cell metabolomics

The use of scRNA‐seq in immunometabolism studies has provided insights into the transcriptional regulation of metabolic pathways in both healthy and diseased states [16, 49, 50, 52, 65]. For example, the application of scRNA‐seq on naive, activated, effector, and memory CD8^+^ T cells identified asparagine synthetase as an important modulator of CD8^+^ T cell differentiation toward an effector T cell phenotype [66]. Especially when combined with other single‐cell technologies, scRNA‐seq can make important contributions to the integration of metabolic and immune heterogeneity. Gubin et al. [67] utilized scRNA‐seq combined with cytometry by time of flight (CyTOF, see Section 2.1.2) to study murine sarcomas treated with immune check point blockade (ICB). By combining these single‐cell techniques, the authors could comprehensively investigate both functional and metabolic changes in response to ICB in intertumoral macrophages. Hence, scRNA‐seq has clear potential for use in immunometabolism research (Table 1): the technique has an unbiased character and can obtain both functional and phenotypical characteristics of the analyzed cells [16, 47]. However, scRNA‐seq has limitations when studying metabolism (Table 1). Most importantly, scRNA‐seq does not provide information on protein expression or activity of metabolic enzymes, as it provides information only at transcriptomic level [68]. Hence, this technique only provides an indication of the immunometabolic status of a cell, and metabolomics (see below) or proteomics studies are needed to verify the metabolic pathway activity.

**Table 1:** Advantages and disadvantages of advanced methods applicable to resolve challenges in studying immunometabolism.

Method (*Used in clinical samples)	Advantages	Disadvantages	Examples
*Challenge 1: Studying immune and metabolic heterogeneity*			
scRNA‐seq*	Unbiased Combined metabolic and phenotypic information	Limited sample size Discordance between mRNA levels and protein levels/protein functionality Indirect method to measure metabolism	[49, 50, 51, 52, 67]
SCM	Low cell numbers needed Direct metabolite measurements High resolution of heterogeneity	Limited number of metabolites covered Costly and time‐consuming Single‐cell isolation is needed, which might affect sample quality and metabolic status	[69, 70]
SCENITH * Met‐Flow	Combined phenotypic and metabolic information Fast Most facilities have the appropriate machinery High‐throughput acquisition and resolution of low‐expressed markers (spectral flow cytometry)	Fluorescent spillover Indirect metabolic measurements Selection of targets needed	[20, 73, 74, 75]
CITE‐seq*	Quantifies cell surface proteins alongside RNA molecules in a single‐cell fashion Applicable for tissue as well as body liquids and cultured cells	Indirect metabolic measurements Selection of the protein targets needed	[77, 78, 79, 80, 81]
Stable isotope tracing	Information about metabolic pathway activity and metabolite sources	Tracers are expensive So far mostly been used in bulk analysis Combinations of tracers require ultra‐high‐resolution equipment	[98, 99, 100, 101, 102, 103, 104, 105]
*Challenge 2: Resolving spatial resolution*			
CyTOF*	High dimensional High throughput Applicable for spatial as well as single‐cell samples Metabolic profile obtained on protein level	Not applicable for weakly expressed markers Limited number of protein markers Advanced biostatistics and bioinformatics needed Indirect metabolic measurement Machinery not available at all research facilities Lower sensitivity compared to flow cytometry	[82, 83]
Imaging MS (MSI), such as MALDI‐MS*	Direct measurements of metabolism *in situ* Retain intact tissue structure Potential to measure a broad range of lipids/metabolites	Must be combined with other histological or morphological information to couple metabolic to phenotypic characteristics Sensitivity for low *m/z* (70–500 Da) remains limited (MALDI) Challenging identification due to high matrix interruption (MALDI) Advanced techniques/instrument are needed, which are not available at all research facilities	[88, 90, 91]
Advanced co‐culture systems	Method to study the metabolic interplay between specific cells Especially suited for studying cell–cell interactions through secreted factors Rapid separation minimizes disruption of the metabolome during isolation Can be personalized by including patient materials Valuable for pathogenetic/mechanistic studies	Separation is needed before single‐cell metabolism studies can be performed Limited in the number of co‐cultured cell types Disregards circulation and lymphoid system Artificial concentrations of nutrients	[92, 93, 94, 95, 96, 97]

**Fig. 2:**
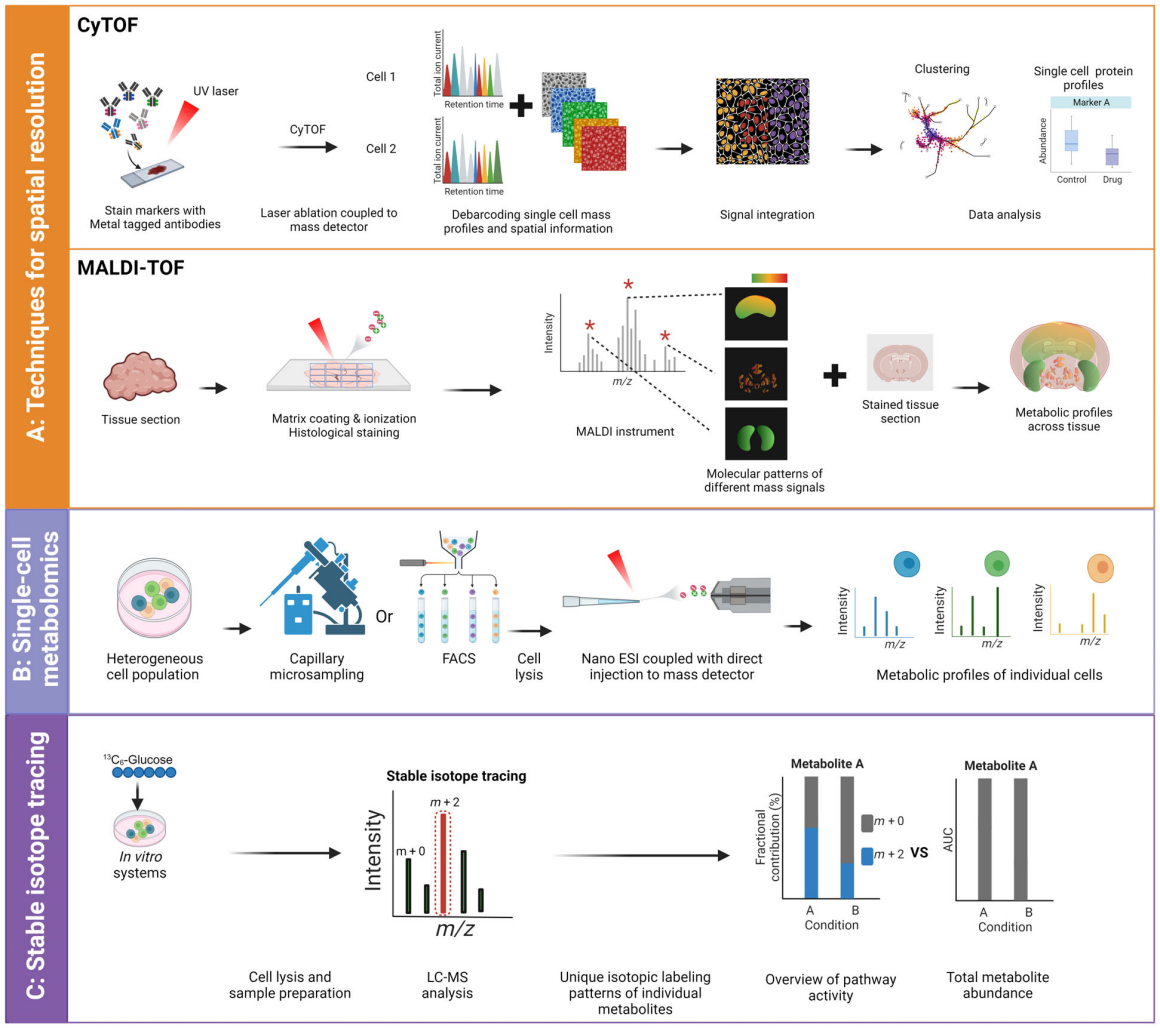
Workflows of selected state‐of‐the‐art methods for studying metabolism. (A) Techniques for spatial resolution. Cytometry by time of flight (CyTOF) representative workflow: protein markers in samples are stained with stable heavy metal‐tagged antibodies, ionized by laser ablation, and analyzed with a mass detector, often TOF. Single‐cell mass profiles are de‐barcoded and combined with spatial information, followed by downstream data analysis and visualization. In MS imaging, for example, in matrix‐assisted laser desorption/ionization (MALDI‐TOF), a tissue section is coated with a matrix for extraction and ionization of metabolites which are subsequently measured with a TOF mass detector. Histological staining can be performed on consecutive tissue sections, to simultaneously allow for immune phenotypic characterization of (immune) cells within the tissue. Data analysis results in spatially resolved mass spectra, which can be combined with histological results. (B) For single‐cell metabolomics (lilac panel), cells are isolated by either direct capillary microsampling or fluorescence‐activated cell sorting (FACS), followed by nano‐electrospray ionization (nano‐ESI) coupled to direct injection into a mass spectrometer, thereby, providing metabolic profiles of individual cells. (C) Unlike steady‐state metabolomics, which only provides metabolite abundances, stable isotope tracing (dark purple panel) provides information on pathway activities. Incubation with tracers of choice, for example, ^13^C_6_‐glucose *in vitro* or *in vivo* is followed by sample collection, metabolite extraction, and mass spectrometry. Data are analyzed as fractional contribution (%) of tracer relative to the total intensity of a given metabolite. Differences in pathway activities in condition A versus B can be derived from differences in isotope fractions, which persist in the absence of differences in total metabolite abundances. Created with Biorender.com.

Metabolomics can profile metabolites in various types of samples, including samples derived from tissues or body fluids. Mass spectrometry (MS), coupled with separation methods such as liquid chromatography (LC) or gas chromatography (GC), is widely used in metabolomic studies. With the rapid development of more sensitive mass spectrometers, single‐cell metabolomics (SCM) is now within reach. In a typical SCM experiment, single cells are isolated using flow cytometry, laser capture microdissection, or micropipette sampling. Subsequently, these isolated cells are lysed and directly infused into the mass spectrometer, resulting in metabolite profiles that provide insights into the metabolic processes occurring in individual cells (Fig. 2B). For example, a study investigating circulating tumor cells (CTC) from gastric and colorectal cancer patients by SCM found clear differences in lipid metabolism, with CTCs from colorectal cancers showing higher levels of acylcarnitine and sterol lipids, while CTCs from gastric cancer showed increased glycerophospholipids [69]. In addition to the measurement of endogenous metabolites, SCM can also be utilized for measuring drug levels in single cells [70]. Bensen et al. were able to measure intracellular levels of the chemotherapeutic drugs *in vitro*, as well as within isolated bladder cancer cells from patients. Interestingly, they detected significant differences in drug uptake by cells, demonstrating the potential of quantitative SCM in precision medicine. SCM methods have many advantages over bulk metabolism methods (Table 1), including the ability to measure metabolic heterogeneity between as well as within individual cell types. However, several disadvantages associated with SCM still exist (Table 1). One of the primary challenges is the dependency on the isolation of single cells, and the isolation method can profoundly affect the sample quality and metabolic status [71, 72]. Moreover, the throughput of SCM is low when compared to bulk LC/GC–MS.

#### 2.1.2 Antibody‐based methods to study metabolism at single‐cell resolution: SCENITH, Met‐Flow, CITE‐seq, and CyTOF

Within the field of immunology, flow cytometry is a fundamental method to evaluate both phenotypical and functional characteristics of immune cells at the single‐cell level. Therefore, utilizing flow cytometry for metabolic profiling of immune cells enables researchers to combine metabolic insights with phenotypic and functional information in a single measurement. To study metabolic regulation, two flow cytometry‐based methods are used: (a) single‐cell energetic metabolism by profiling translation inhibition (SCENITH); and (b) high parameter method based on antibodies against rate‐limiting metabolic enzymes, referred to as Met‐Flow [73, 74]. SCENITH combines inhibitors of metabolic enzymes with puromycin. The latter is incorporated as a measure for protein synthesis and followed by adding a monoclonal anti‐puromycin antibody. As protein synthesis serves as a readout for global metabolic activity, this technique allows us to determine the dependency of cells on specific metabolic pathways. In contrast, Met‐Flow uses fluorophore‐labeled antibodies against important metabolic enzymes or cell surface transporters together with antibodies against lineage‐specific makers. In recent years, SCENITH has been used to determine the metabolic regulation of cultured cells, as well as tumor biopsies and blood samples [73]. Adamik et al. [75] used SCENITH to describe the metabolic differences between mature and vitamin D3‐induced tolerogenic DCs (tolDCs). In addition, the metabolic profile generated by SCENITH has been successfully linked to single‐cell metabolic gene expression data of renal carcinomas and juxta‐tumoral tissues, demonstrating that SCENITH can contribute to metabolism studies in a multi‐omics fashion [68]. Flow cytometry‐based methods to study metabolism offer many advantages (Table 1). Information about metabolic heterogeneity can be coupled to phenotypic and functional information at the single‐cell level. As flow cytometry methods are easily scalable, they are applicable for measuring rare immune cell populations. However, flow cytometry‐based methods can only provide an indirect measurement of cellular metabolism. For example, Met‐Flow solely measures up‐ or down‐regulation of metabolic enzymes or transporters, and not enzyme activity or (relative) metabolite levels. Moreover, the availability of suitable antibodies for metabolic targets, as well as the limitation to 40 markers on flow cytometry, restricts the scope of the research to specific predetermined pathways (Table 1) [76].

Recently, another interesting antibody‐based technique has emerged: cellular indexing of transcriptomes and epitopes by sequencing (CITE‐seq) that combines scRNA‐seq with antibody‐based measurements of protein expression levels [77]. Instead of regular antibodies utilized for flow cytometry, CITE‐seq makes use of antibodies with unique barcodes. This allows for quantification of cell surface proteins alongside RNA molecules in a single‐cell fashion. CITE‐seq can be applied to tissues as well as body liquids and cultured cells, making this technique applicable for clinical use [56, 77, 78, 79, 80, 81] (Table 1). In recent years, CITE‐seq has been applied to uncover the immune landscape of several diseases, including multiple cancer types [79, 80, 81] and COVID‐19 [56, 78]. As both scRNA‐seq and flow cytometry‐based metabolic techniques have shown to be advantageous for unraveling immunometabolism at the single‐cell level, combining these techniques through CITE‐seq shows immense potential in future immunometabolism research.

Finally, CyTOF‐based methods with antibody panels focusing on metabolic enzymes have been crucial in validating metabolic phenotypes in their original tissue contexts (Fig. 2A). In a CyTOF experiment, protein markers in samples are stained with stable heavy metal‐tagged antibodies, followed by ionization by laser ablation and analysis with a mass detector. Single‐cell mass profiles are subsequently de‐barcoded. When performed on tissue slices, CyTOF generates a spatial overview of the distribution of metabolic and immunologic proteins (Fig. 2A). As CyTOF is applicable to both suspension cells and tissue samples, it shows great potential in spatially resolving both cellular heterogeneity and metabolic heterogeneity. Hartmann et al. [82] developed a CyTOF method called single‐cell metabolomic regulome profiling (scMEP), which utilizes antibodies against immunological and metabolic proteins to determine both immune cell phenotypes and metabolic pathway activities. Using scMEP, CD39^+^ PD1^+^ T cells were found to be spatially restricted to the tumor‐immune boundary in human colorectal cancer [82]. Levine et al. [83] also implemented CyTOF in studying the metabolic regulators of isolated CD8^+^ T cells post‐*Listeria monocytogenes* infection *in vivo* and found elevation of proteins in both glycolysis and oxidative phosphorylation. Altogether, these studies illustrate the potential of CyTOF in acquiring spatial information for studying both immune cells isolated from tissues and tissue slices. Despite the many advantages of CyTOF‐based methods (Table 1), several disadvantages remain (Table 1). These include limitations on the amount of protein markers that can be measured simultaneously and the indirect nature of the metabolic measurements via metabolic protein expression levels. Moreover, CyTOF‐based methods are not suitable for weakly expressed markers and exhibit lower sensitivity compared to flow cytometry [16].

### 2.2 Understanding the spatial heterogeneity of the metabolome

Mass spectrometry imaging (MSI) is currently one of the most prominent techniques for studying spatial metabolomics *in situ*. Combining the spatial information of imaging with mass spectrometry allows direct measurements of samples in their native state, providing insights into the spatial regulation of metabolism within cells and tissues [84, 85]. With MSI, metabolites can be ionized using different ionization techniques, such as secondary ion mass spectrometry (SIMS), desorption electrospray ionization (DESI), infrared matrix‐assisted laser desorption electrospray ionization (IR‐MALDESI), or matrix‐assisted laser desorption/ionization (MALDI; Fig. 2A) [84, 85, 86]. In MALDI, for example, a tissue section is coated with a matrix, and after extraction and ionization of metabolites with a laser pulse, a mass spectrum is recorded. By scanning a tissue section in this manner, spatially resolved mass spectra are recorded, which can be combined with histological staining of the same tissue section (Fig. 2A). The type of ionization technique and mass analyzer determines the biomolecules analyzed, sensitivity, and spatial resolution achieved.

When coupled with other imaging methods such as immunohistochemistry or immunofluorescence microscopy, MSI is a powerful method to spatially resolve metabolic and functional heterogeneity. For instance, by combining MSI and immunohistochemistry, Greco et al. [87] uncovered multiple plaque‐resident macrophages with distinct lipid signatures as well as different phenotypical and localization characteristics in murine atherosclerotic plaques. Rappez et al. [88] developed SpaceM, by combining MALDI with light microscopy, to capture the spectrum of metabolic rewiring in mono‐ and co‐culture models *in vitro* with single‐cell resolution. Here, human hepatocytes, stimulated with fatty acids, differentiated into subpopulations with unique metabolic states. Notably, Holzlechner et al. demonstrated the potential of MSI for visualizing the distribution of different immune cell subsets in colon tissue, for the first time. The authors found that certain metabolites were specifically localized in the lymphoid follicular structures or lamina propria, where they also observed CD3 and CD206 expression, respectively [89]. Finally, studies on cancer cells show that MSI has the potential to reveal disease‐relevant metabolic states. Using MSI, Cuypers et al. [90] described the metabolic and molecular profiles of multiple human breast cancer cell lines at single‐cell and subcellular levels. These data resulted in a recognition model that can be used to diagnose breast cancer subtypes in complex tissue sections, highlighting the diagnostic value of MSI. Currently, MSI still deals with some drawbacks, including low sensitivity for low mass ranges (70–500) and matrix interruption which hinders compound identification (Table 1) [88, 90, 91]. However, with rapid advances in spatial single‐cell MSI instrumentation and analysis pipelines, we foresee that this technique will complement single‐cell sequencing techniques in the future, revealing correlations between cellular phenotypes and spatial metabolic phenotypes/characteristics.

### 2.3 Development of advanced model systems to map metabolic crosstalk

#### 2.3.1 Model systems to resolve cell–cell metabolic interactions

*In vitro* model systems with controllable parameters have advanced the field of immunometabolism. Although lacking the complexity of *in vivo* cell–cell interactions, *in vitro* cell‐culture systems allow for manipulation of specific cell types or immune subsets. More sophisticated models incorporating several cell types, such as Transwell systems, organ‐on‐a‐chip models, or organoids, offer opportunities to study metabolic crosstalk in direct (i.e., with cell–cell contact) and indirect co‐cultures, facilitating pathogenetic and mechanistic studies. These models can also be personalized by including patient materials (Table 1). Transwell co‐culture systems are especially suited for studying cell–cell interactions mediated by secreted factors. Moreover, they allow rapid separation of cells for metabolite extraction, which minimizes metabolome disruption during cell isolation. Rabold et al. [92] performed transcriptomics, metabolomics, and lipidomics to study interactions in Transwell co‐culture of peripheral blood monocytes and thyroid cancer cells. They found that co‐culture with thyroid cancer cells stimulates lipid synthesis in monocytes, which in turn promotes secretion of tumor necrosis factor‐α and Interleukin‐6 (IL‐6) and increases the production of reactive oxygen species.

Engineered TME‐mimicking culture systems, such as tumor/organ‐on‐the‐chip, recapitulate key characteristics of the *in vivo* microenvironments through the preservation of tissue mechanics, cellular compositions, and matrix signals. Several systems that include immune cells as components have been tested in recent years [93, 94]. For example, Trapecar et al. [93] investigated the progression of inflammatory bowel disease and its connection to liver diseases using a combination of microphysiological systems of the gut and liver, along with Th17 and regulatory T cells as immune components. Additionally, Bein et al. [94] used an intestine‐on‐a‐chip microfluidic culture device to investigate the effect of coronavirus infection on the human intestine. 3D co‐culture models such as organoids have been established to maintain microenvironment complexity and provide novel platforms to study multicellular metabolic crosstalk [95, 96]. For instance, *in vivo* metabolic features of original tissue have been shown to be reproduced in 3D spheroids freshly isolated from kidney tissue [97].

#### 2.3.2 Advances in stable isotope tracing to map metabolic reactions *in vitro*

Both bulk and single‐cell metabolomics generate snapshots of metabolite levels in (single) cells. However, the levels of intermediates within a specific metabolic pathway do not always correlate with the activity of that pathway [16, 66]. In stable isotope tracing experiments, cells or tissues are incubated with stable isotopically labeled nutrients, leading to incorporation of the stable isotope in downstream metabolites. Subsequently, labeled metabolites are measured by GC/LC–MS (Fig. 2C), providing information on pathway dynamics in cell populations (Table 1). Stable isotope tracing has been pivotal in studying pathway dynamics and understanding metabolic switches in many immune cell types [98, 99, 100, 101, 102, 103, 104, 105]. For example, Mensink et al. [100] recently performed tracing experiments using ^13^C_6_‐glucose and ^13^C_5_‐glutamine to determine the effect of tumor necrosis factor receptor 2 (TNRF2) co‐stimulation on CD4^+^ conventional and regulatory T cells. TNFR2 induced upregulation of glutamine metabolism in both cell types, while enhanced glucose metabolism was only observed in regulatory T cells. ^13^C‐tracing studies have also been used to link metabolic regulation to immune cell functionality. For example, Siska et al. [99] combined tracing studies with flow cytometry to determine the effect of d‐/l‐kynurenine on T cell functionality and metabolism. d‐/l‐kynurenine induced apoptosis in T cells, paired with reduced levels of free fatty acids. To determine whether the lower levels of free fatty acid levels were a result of reduced fatty acid synthesis or elevated fatty acid oxidation, the authors employed ^13^C‐labeled glucose, glutamine, or palmitate for stable isotope tracing. Their data indicated that d‐/l‐kynurenine exposure resulted in increased palmitate incorporation in the TCA cycle, demonstrating increased fatty acid oxidation. At the same time, no differences were observed in glucose and glutamine incorporation in fatty acids, ruling out the involvement of fatty acid synthesis in the observed phenotype.

Combining isotope tracing with advanced *in vitro* model systems enables mapping of metabolic crosstalk in the context of a more complex heterogeneous (tissue) architecture. Curtis et al. pre‐labeled the glycogen store of ovarian cancer cells with ^13^C_6_‐glucose and subsequently traced this labeled glycogen in a Transwell co‐culture with cancer‐associated fibroblasts. Using this system, the authors found that cytokines (IL‐6) and chemokines (CCL5, CXCL10) secreted by cancer‐associated fibroblasts induce glycogen breakdown in ovarian cancer cells to promote proliferation and invasion [106]. Using ^13^C_6_‐glucose tracing in combination with a Transwell co‐culture system, Matamala Montoya et al. [101] recently demonstrated the importance of bone‐marrow stromal cells in inducing drug resistance‐associated metabolic rewiring in multiple myeloma. To map cell‐type‐specific dynamic changes in central carbon metabolism, Wang et al. [107] applied an advanced high‐spatial‐resolution metabolomics approach using MALDI‐MSI combined with isotope tracing in an organoid co‐culture. While these examples are not directly linked to immune cells, they accentuate the potential of combining advanced (co‐)culture models with stable isotope tracing to understand immunometabolic crosstalk. Disadvantages of stable isotope tracing include their high costs (Table 1). Moreover, information is restricted depending on the stable isotope tracers that are used. Also, when combining tracers, ultra‐high‐resolution equipment is needed.

## 3 Immunometabolism in clinical practice

**Fig. 3:**
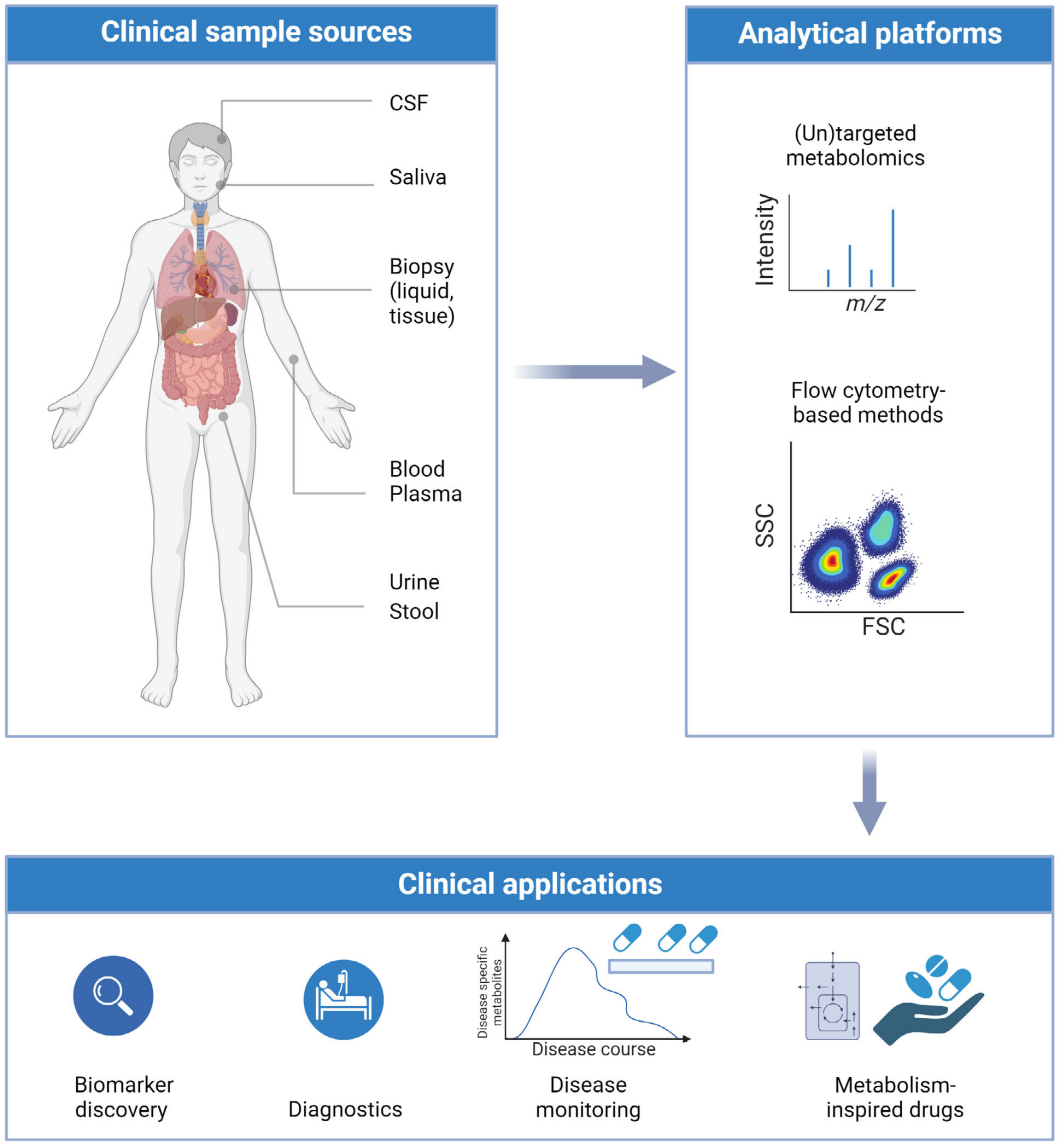
Immunometabolism techniques and their clinical applications. Increasing metabolic characterization of various sample types can yield important insights into the development of metabolism‐inspired biomarkers and drug delivery platforms, and improving therapeutics. Moreover, this increased metabolic characterization can aid disease monitoring and the assessment of treatment efficacy. Sample material for metabolomics research in the clinic includes various body fluids, such as plasma, whole blood, urine saliva, and cerebrospinal fluid (CSF), as well as tissue slices, stool, and biopsies from various tissues. These samples can be analyzed via different techniques including, (un)targeted metabolomics or flow cytometry‐based metabolic methods. Created with Biorender.com.

Due to the importance of cellular metabolism in development and progression of several diseases, metabolomics analyses have acquired a prominent position in current clinical research [32, 108, 109, 110, 111, 112]. Different methods to study cellular metabolism are currently used for identifying new drug targets and biomarkers, as well as for diagnostic and prognostic purposes (Fig. 3). For diagnostic purposes, body fluids, such as blood or urine, that can be obtained with minimally invasive procedures are often used. However, metabolite measurements can also be performed on tissue biopsies, tissue slices, and cerebrospinal fluids (Fig. 3). Measurements are often targeted, only measuring a few metabolites per sample. The benefits of such targeted metabolomics include analysis of relevant metabolic pathways, optimized sample preparation, reliable identification, absolute quantification based on standards, and the possibility to filter out analytical artifacts [113]. For example, Jacob et al. developed a targeted LC–MS method to measure 220 metabolites associated with a range of inborn errors of metabolism (IEMs) in a single assay, introducing an attractive diagnostic method for these disorders. More recently, methods that assess the metabolome in an unbiased fashion, so‐called untargeted metabolomics, have been developed. These methods are specifically used in discovering novel biomarkers, which can be used in clinical settings in the future. For instance, Coene et al. [114] developed a “next‐generation metabolic screening” technology, which uses high‐resolution LC–MS to profile the plasma metabolome in prospective IEM patients in an unbiased manner. Not only can untargeted metabolomics extend the coverage and scope of metabolites detected, but modern high‐resolution technologies entail enhanced accuracy in measurements [114]. In addition to the diagnostic applications, metabolomics has also contributed to the identification of novel biomarkers for IEMs (reviewed in Ref. [115]). Despite the many advantages of untargeted metabolomics, difficulties in confident identification of (novel) metabolites persist.

High throughput is a key factor in clinical research. Flow cytometry‐based methods are, therefore, especially suited for clinical immunometabolism research (Fig. 3). Currently, studies in clinical settings either focus on immune cell phenotypes by measuring cell surface markers with flow cytometry or measuring metabolite levels by, for example, LC–MS. Recently, SCENITH was used to study the differences in metabolic regulation between CD4^+^ T cells in blood of healthy volunteers and patients with COVID‐19 acute respiratory distress syndrome [116]. Using SCENITH, the authors coupled metabolic differences between patients and healthy controls to their respective immune phenotypes. The number of flow cytometry‐based immunometabolism studies from clinical research is still limited. However, the study by Karagiannis et al. [116] effectively demonstrates the potential of this method in uncovering the metabolic regulation of immune cells in human inflammatory diseases, as well as the possibility of combining it with other metabolic methods. Despite these advances, the standard application of immunometabolism techniques in clinical research and practice persists to be challenging, as specialized equipment and trained personnel are needed for measurement and data analysis.

## 4 Conclusions

**Fig. 4:**
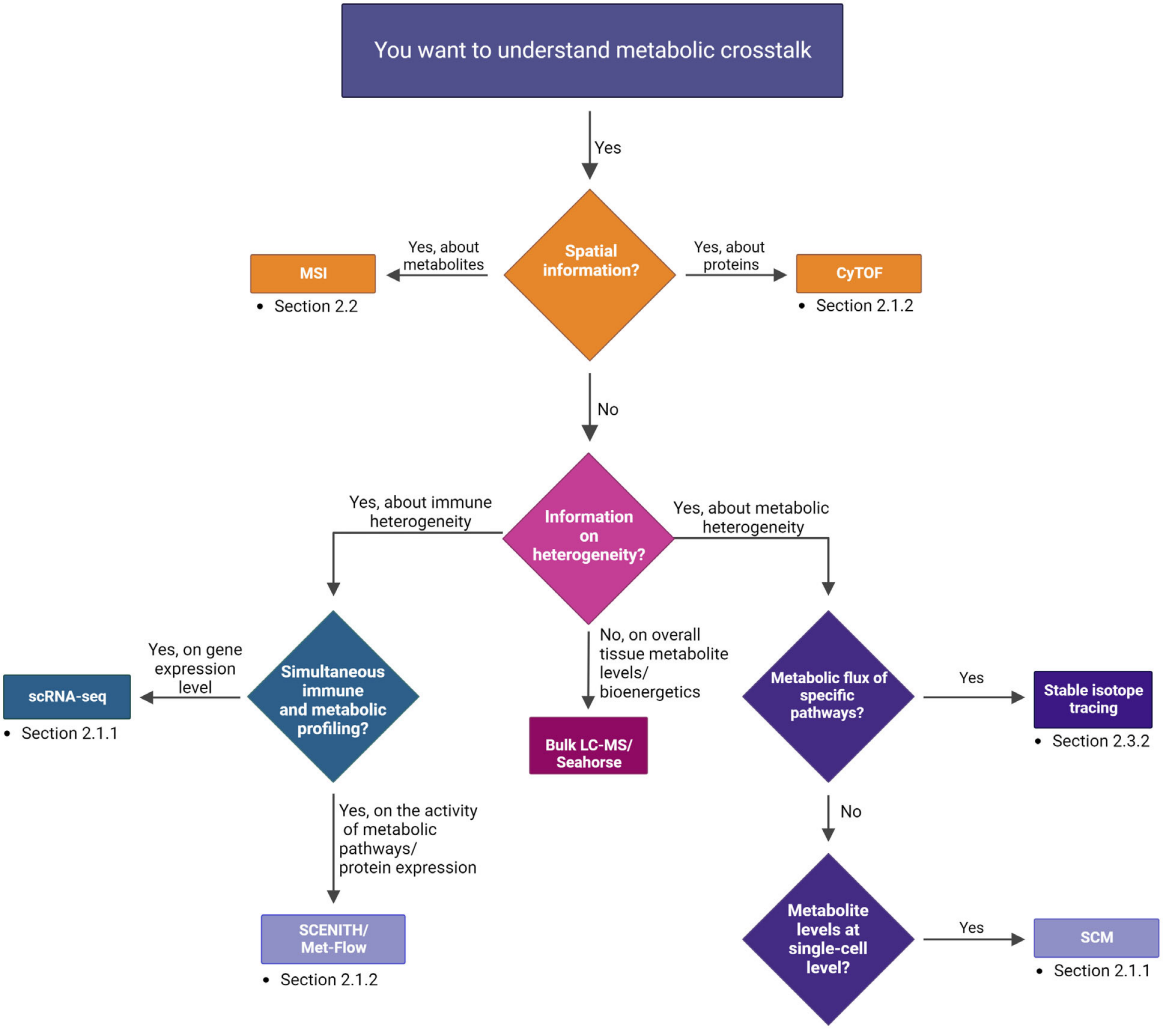
Flowchart displaying different techniques to study heterogeneity in an immunometabolic context. Starting with the need to tackle heterogeneity, the reader finds both cytometry by time of flight (CyTOF) and mass spectrometry imaging (MSI) as options to study spatial heterogeneity within a sample. When interested in studying immune heterogeneity, flow cytometry‐based methods (Met‐flow and single‐cell energetic metabolism by profiling translation inhibition (SCENITH)) or single‐cell RNA sequencing (scRNA‐seq) are suggested, which can both give information on the expression of metabolic proteins, at both protein and RNA levels, respectively. Finally, stable isotope tracing and single‐cell metabolomics (SCM) are proposed as methods to study metabolic heterogeneity at a metabolite level. Created with Biorender.com.

Maintaining immune homeostasis requires synchronized and fine‐tuned metabolic crosstalk at cellular, tissue, and organ levels. In metabolic crosstalk, metabolites not only serve as rapid messengers and signaling molecules that reflect the real‐time metabolic state of cells but also act through various communication modules in both local and distal sites. Decoding these messages is facilitated by the emergence and integration of novel technologies. Advancements in MS methods, for instance, SCM [117, 118, 119] and computational pipelines of spatial SCM [53], present solutions to untangle the complexity of metabolic crosstalk. Moreover, flow cytometry‐based characterization, CyTOF, stable isotope tracing, and sophisticated co‐culture methods all provide unique information to further understand metabolic crosstalk in the context of the microenvironment. Selecting the appropriate technique for studying metabolic crosstalk involves considering several factors. To assist readers in identifying the most suitable method for their research, we have created a flowchart outlining the key techniques discussed in this review (Fig. 4). This flowchart guides the reader through distinct levels of heterogeneity (e.g., spatial, metabolic, and immunological). Although we categorize these techniques according to the primary challenge they address, most of them can be utilized for multiple challenges. For example, CyTOF‐based methods can aid our understanding of cellular heterogeneity at the cellular and spatial levels.

To obtain a complete overview of the various levels of heterogeneity, several (omics) techniques should be combined. Especially the integration of single‐cell or spatial metabolomics with proteomics, transcriptomics, and genomics holds promise in advancing our knowledge of immunometabolism at a systems biology level. In addition, a cell itself is also an interesting microenvironment that provides a platform for inter‐organelle metabolic communication. The advances in subcellular metabolomics, although still in its infancy, hold promise for further investigation on this type of crosstalk [120]. Currently, most stable isotope tracing studies are performed using targeted metabolomics. In recent years, several groups have shown the potential of combining untargeted metabolomics with stable isotope tracing [121, 122]. For example, Puchalska et al. [122] combined ^13^C_6_‐glucose tracing with untargeted LC–MS to unravel the differences in glucose metabolism for different polarization states of macrophages. Nevertheless, when performing untargeted metabolomics, the certainty in metabolite identification remains a major issue, as spectral databases only exhibit limited amounts of reference spectra. Therefore, in the metabolomics field, uploading reference spectra to databases, such as The Global Natural Product Social Molecular Networking (GNPS), should become a priority [123]. For single‐cell metabolomics, focus should be on optimization of cell isolation procedures as well as increasing resolution and throughput to allow for reliable metabolite identification [119].

Similar to mass spectrometry‐based techniques, flow cytometry‐based techniques still have some hurdles to overcome. Currently, the limitation to markers restricts flow cytometry‐based methods to the analysis of specifically chosen pathways, limiting the scope of the research (Table 1) [68]. A promising development to overcome this restriction is the introduction of spectral flow cytometry. In contrast to conventional flow cytometry, in which filters are used to measure only a selection of the signal of a fluorophore, spectral flow cytometry measures the signal of a fluorophore over the entire spectra of all the lasers, resulting in a specific spectral fingerprint for each fluorophore. This development potentially allows scientists to increase the number of markers that can be measured within one sample. Using this technology, Heieis et al. [20] elucidated the spectra of eleven metabolic proteins of murine tissue macrophages from multiple peripheral organs and within populations from one anatomical site.

Our understanding of immune cell adaptations and potential metabolic crosstalk within different tissues is restricted to limited pathways. How adaptations are controlled at a global scale warrants further investigation. In contrast to flow cytometry‐based techniques, scRNA‐seq is not restricted to predetermined markers. However, most current scRNA‐seq studies do not focus on metabolic data generated by this technique and metabolic enzymes are weighed down by other cellular processes. Filtering on metabolic enzymes can thus shed light on existing scRNA‐seq datasets, as demonstrated by Artyomov et al. [16], which exemplified the potential of mining previously generated scRNA‐seq datasets to increase our knowledge on transcriptomic regulation of metabolism in immune cells.

Moreover, the usage of advanced (co‐)culture models will enhance our understanding of immunometabolic interactions in model systems, which more closely represent *in vivo* microenvironments. For example, organoids can be combined with Transwell systems to study metabolic crosstalk. Such a set‐up would retain tissue features, while also facilitating separation of specific cell types. Likewise, direct tumor slides may also be added into microfluidic devices, as shown in drug screening in murine brain tissues [124]. Finally, Chen et al. [125] applied fluorescence microscopy to guide the live single‐cell sampling for MS‐based SCM in a direct co‐culture of drug‐resistant and drug‐sensitive colorectal cancer cells. This live single‐cell sampling strategy avoids metabolic disturbance associated with fluorescence‐activated cell sorting (FACS) and reveals that co‐culture induces drug‐resistance and metabolic rewiring in drug‐sensitive cells.

In conclusion, here, we discussed the important challenges in immunometabolism research, highlighted the state‐of‐the‐art techniques suited to address these challenges, and indicated advancements that can push the field forward. The parallels drawn between cancer metabolism and immunometabolism suggest mutual benefits from shared discoveries and methodological advancements. Integration of knowledge in both cancer metabolism and immunometabolism is, therefore, essential for future research. We envision that technological advancements in experimental and computational biology will provide more breadth (spatial and untargeted), depth (single‐cell, isotopic characterization), and comprehensive (relevant models and multi‐omics) resolution of metabolic crosstalk in healthy and diseased conditions. This will facilitate metabolism‐targeted personalized and precision medicine, and improve therapeutics for patients suffering from inflammatory diseases and cancers alike.

## Conflict of interest

The authors declare no conflict of interest.

## Author contributions

FWMV, TNMT, JCC, EAZ and CRB conceptualized the study. FWMV and TNMT conducted the literature review, generated the figures and drafted the manuscript. JCC, EAZ, FB and CRB critically reviewed the manuscript, which was edited by FWMV, EAZ and CRB. All authors approved the final version of the manuscript. [Correction added on 01 April 2024, after first online publication: The Author contributions have been expanded and clarified in this version.]

## References

[1] Cao Y , Rathmell JC , Macintyre AN . Metabolic reprogramming towards aerobic glycolysis correlates with greater proliferative ability and resistance to metabolic inhibition in CD8 versus CD4 T cells. PLoS One. 2014;9(8):e104104. 25090630 10.1371/journal.pone.0104104 PMC4121309

[2] Wang R , Dillon CP , Shi LZ , Milasta S , Carter R , Finkelstein D , et al. The transcription factor Myc controls metabolic reprogramming upon T lymphocyte activation. Immunity. 2011;35(6):871–882. 22195744 10.1016/j.immuni.2011.09.021 PMC3248798

[3] Greiner EF , Guppy M , Brand K . Glucose is essential for proliferation and the glycolytic enzyme induction that provokes a transition to glycolytic energy production. J Biol Chem. 1994;269(50):31484–31490. 7989314

[4] Ardawi MS . Glutamine and glucose metabolism in human peripheral lymphocytes. Metabolism. 1988;37(1):99–103. 3336288 10.1016/0026-0495(88)90036-4

[5] Leone RD , Powell JD . Metabolism of immune cells in cancer. Nat Rev Cancer. 2020;20(9):516–531. 32632251 10.1038/s41568-020-0273-y PMC8041116

[6] Pearce EJ , Everts B . Dendritic cell metabolism. Nat Rev Immunol. 2015;15(1):18–29. 25534620 10.1038/nri3771 PMC4495583

[7] O'Sullivan D , Pearce EL . Targeting T cell metabolism for therapy. Trends Immunol. 2015;36(2):71–80. 25601541 10.1016/j.it.2014.12.004 PMC4323623

[8] Chen W , Liang X , Peterson AJ , Munn DH , Blazar BR . The indoleamine 2,3‐dioxygenase pathway is essential for human plasmacytoid dendritic cell‐induced adaptive T regulatory cell generation 1. J Immunol. 2008;181:5396–5404. 18832696 10.4049/jimmunol.181.8.5396 PMC2614675

[9] Fallarino F , Grohmann U , You S , McGrath BC , Cavener DR , Vacca C , et al. The combined effects of tryptophan starvation and tryptophan catabolites down‐regulate T cell receptor ζ‐chain and induce a regulatory phenotype in naive T cells. J Immunol. 2006;176(11):6752–6761. 16709834 10.4049/jimmunol.176.11.6752

[10] Caputa G , Castoldi A , Pearce EJ . Metabolic adaptations of tissue‐resident immune cells. Nat Immunol. 2019;20:793–801. 31213715 10.1038/s41590-019-0407-0

[11] Lavin Y , Winter D , Blecher‐Gonen R , David E , Keren‐Shaul H , Merad M , et al. Tissue‐resident macrophage enhancer landscapes are shaped by the local microenvironment. Cell. 2014;159(6):1312–1326. 25480296 10.1016/j.cell.2014.11.018 PMC4437213

[12] Pan Y , Tian T , Park CO , Lofftus SY , Mei S , Liu X , et al. Survival of tissue‐resident memory T cells requires exogenous lipid uptake and metabolism. Nature. 2017;543(7644):252–256. 28219080 10.1038/nature21379 PMC5509051

[13] Davies LC , Rice CM , Palmieri EM , Taylor PR , Kuhns DB , McVicar DW . Peritoneal tissue‐resident macrophages are metabolically poised to engage microbes using tissue‐niche fuels. Nat Commun. 2017;8(1):2074. 29234000 10.1038/s41467-017-02092-0 PMC5727035

[14] Crowl JT , Heeg M , Ferry A , Milner JJ , Omilusik KD , Toma C , et al. Tissue‐resident memory CD8+ T cells possess unique transcriptional, epigenetic and functional adaptations to different tissue environments. Nat Immunol. 2022;23(7):1121–1131. 35761084 10.1038/s41590-022-01229-8 PMC10041538

[15] T'Jonck W , Guilliams M , Bonnardel J . Niche signals and transcription factors involved in tissue‐resident macrophage development. Cell Immunol. 2018;330:43–53. 29463401 10.1016/j.cellimm.2018.02.005 PMC6108424

[16] Artyomov MN , Van den Bossche J . Immunometabolism in the single‐cell era. Cell Metab. 2020;32:710–725. 33027638 10.1016/j.cmet.2020.09.013 PMC7660984

[17] Gautier EL , Shay T , Miller J , Greter M , Jakubzick C , Ivanov S , et al. Gene‐expression profiles and transcriptional regulatory pathways that underlie the identity and diversity of mouse tissue macrophages. Nat Immunol. 2012;13(11):1118–1128. 23023392 10.1038/ni.2419 PMC3558276

[18] Gosselin D , Link VM , Romanoski CE , Fonseca GJ , Eichenfield DZ , Spann NJ , et al. Environment drives selection and function of enhancers controlling tissue‐specific macrophage identities. Cell. 2014;159(6):1327–1340. 25480297 10.1016/j.cell.2014.11.023 PMC4364385

[19] Schneider C , Nobs SP , Kurrer M , Rehrauer H , Thiele C , Kopf M . Induction of the nuclear receptor PPAR‐γ by the cytokine GM‐CSF is critical for the differentiation of fetal monocytes into alveolar macrophages. Nat Immunol. 2014;15(11):1026–1037. 25263125 10.1038/ni.3005

[20] Heieis GA , Patente TA , Almeida L , Vrieling F , Tak T , Perona‐Wright G , et al. Metabolic heterogeneity of tissue‐resident macrophages in homeostasis and during helminth infection. Nat Commun. 2023;14(1):5627. 37699869 10.1038/s41467-023-41353-z PMC10497597

[21] Sakai M , Troutman TD , Seidman JS , Ouyang Z , Spann NJ , Abe Y , et al. Liver‐derived signals sequentially reprogram myeloid enhancers to initiate and maintain Kupffer cell identity. Immunity. 2019;51(4):655–670.e8. 31587991 10.1016/j.immuni.2019.09.002 PMC6800814

[22] Assmann N , O'Brien KL , Donnelly RP , Dyck L , Zaiatz‐Bittencourt V , Loftus RM , et al. Srebp‐controlled glucose metabolism is essential for NK cell functional responses. Nat Immunol. 2017;18(11):1197–1206. 28920951 10.1038/ni.3838

[23] Bonnardel J , T'Jonck W , Gaublomme D , Browaeys R , Scott CL , Martens L , et al. Stellate cells, hepatocytes, and endothelial cells imprint the Kupffer cell identity on monocytes colonizing the liver macrophage niche. Immunity. 2019;51(4):638–654.e9. 31561945 10.1016/j.immuni.2019.08.017 PMC6876284

[24] Ye J , Fan J , Venneti S , Wan YW , Pawel BR , Zhang J , et al. Serine catabolism regulates mitochondrial redox control during hypoxia. Cancer Discov. 2014;4(12):1406–1417. 25186948 10.1158/2159-8290.CD-14-0250 PMC4258153

[25] Lyons SA , Chung WJ , Weaver AK , Ogunrinu T , Sontheimer H . Autocrine glutamate signaling promotes glioma cell invasion. Cancer Res. 2007;67(19):9463–9471. 17909056 10.1158/0008-5472.CAN-07-2034 PMC2045073

[26] Kandasamy P , Gyimesi G , Kanai Y , Hediger MA . Amino acid transporters revisited: new views in health and disease. Trends Biochem Sci. 2018;43(10):752–789. 30177408 10.1016/j.tibs.2018.05.003

[27] Kelly B , Pearce EL . Amino assets: how amino acids support immunity. Cell Metab. 2020;32(2):154–175. 32649859 10.1016/j.cmet.2020.06.010

[28] Vettore L , Westbrook RL , Tennant DA . New aspects of amino acid metabolism in cancer. Br J Cancer. 2020;122(2):150–156. 31819187 10.1038/s41416-019-0620-5 PMC7052246

[29] Canavan M , Marzaioli V , McGarry T , Bhargava V , Nagpal S , Veale DJ , et al. Rheumatoid arthritis synovial microenvironment induces metabolic and functional adaptations in dendritic cells. Clin Exp Immunol. 2020;202(2):226–238. 32557565 10.1111/cei.13479 PMC7597596

[30] Weyand CM , Goronzy JJ . Immunometabolism in early and late stages of rheumatoid arthritis. Nat Rev Rheumatol. 2017;13:291–301. 28360422 10.1038/nrrheum.2017.49 PMC6820517

[31] Rhoads JP , Major AS , Rathmell JC . Fine tuning of immunometabolism for the treatment of rheumatic diseases. Nat Rev Rheumatol. 2017;13:313–320. 28381829 10.1038/nrrheum.2017.54 PMC5502208

[32] Xu L , Chang C , Jiang P , Wei K , Zhang R , Jin Y , et al. Metabolomics in rheumatoid arthritis: advances and review. Front Immunol. 2022;13:961708. 36032122 10.3389/fimmu.2022.961708 PMC9404373

[33] Qiu J , Wu B , Goodman SB , Berry GJ , Goronzy JJ , Weyand CM . Metabolic control of autoimmunity and tissue inflammation in rheumatoid arthritis. Front Immunol. 2021;12:652771. 33868292 10.3389/fimmu.2021.652771 PMC8050350

[34] Harber KJ , de Goede KE , Verberk SGS , Meinster E , de Vries HE , van Weeghel M , et al. Succinate is an inflammation‐induced immunoregulatory metabolite in macrophages. Metabolites. 2020;10(9):1–14. 10.3390/metabo10090372 PMC7569821 32942769

[35] Peng M , Yin N , Chhangawala S , Xu K , Leslie CS , Li MO . Aerobic glycolysis promotes T helper 1 cell differentiation through an epigenetic mechanism. Science. 2016;354(6311):481–484. 27708054 10.1126/science.aaf6284 PMC5539971

[36] Tannahill GM , Curtis AM , Adamik J , Palsson‐Mcdermott EM , McGettrick AF , Goel G , et al. Succinate is an inflammatory signal that induces IL‐1β through HIF‐1α. Nature. 2013;496(7444):238–242. 23535595 10.1038/nature11986 PMC4031686

[37] Infantino V , Iacobazzi V , Palmieri F , Menga A . ATP‐citrate lyase is essential for macrophage inflammatory response. Biochem Biophys Res Commun. 2013;440(1):105–111. 24051091 10.1016/j.bbrc.2013.09.037

[38] Zhao H , Teng D , Yang L , Xu X , Chen J , Jiang T , et al. Myeloid‐derived itaconate suppresses cytotoxic CD8+ T cells and promotes tumour growth. Nat Metab. 2022;4(12):1660–1673. 36376563 10.1038/s42255-022-00676-9 PMC10593361

[39] Chen YJ , Li GN , Li XJ , Wei LX , Fu MJ , Cheng ZL , et al. Targeting IRG1 reverses the immunosuppressive function of tumor‐associated macrophages and enhances cancer immunotherapy. Sci Adv. 2023;9(17):eadg0654. 37115931 10.1126/sciadv.adg0654 PMC10146892

[40] Aso K , Kono M , Kanda M , Kudo Y , Sakiyama K , Hisada R , et al. Itaconate ameliorates autoimmunity by modulating T cell imbalance via metabolic and epigenetic reprogramming. Nat Commun. 2023;14(1):984. 36849508 10.1038/s41467-023-36594-x PMC9970976

[41] Fischer K , Hoffmann P , Voelkl S , Meidenbauer N , Ammer J , Edinger M , et al. Inhibitory effect of tumor cell–derived lactic acid on human T cells. Blood. 2007;109(9):3812–3819. 17255361 10.1182/blood-2006-07-035972

[42] Pucino V , Certo M , Bulusu V , Cucchi D , Goldmann K , Pontarini E , et al. Lactate buildup at the site of chronic inflammation promotes disease by inducing CD4+ T cell metabolic rewiring. Cell Metab. 2019;30(6):1055–1074.e8. 31708446 10.1016/j.cmet.2019.10.004 PMC6899510

[43] Baardman J , Verberk SGS , Prange KHM , van Weeghel M , van der Velden S , Ryan DG , et al. A defective pentose phosphate pathway reduces inflammatory macrophage responses during hypercholesterolemia. Cell Rep. 2018;25(8):2044–2052.e5. 30463003 10.1016/j.celrep.2018.10.092

[44] Alves TC , Pongratz RL , Zhao X , Yarborough O , Sereda S , Shirihai O , et al. Integrated, step‐wise, mass‐isotopomeric flux analysis of the TCA cycle. Cell Metab. 2015;22(5):936–947. 26411341 10.1016/j.cmet.2015.08.021 PMC4635072

[45] Palsson‐Mcdermott EM , Curtis AM , Goel G , Lauterbach MAR , Sheedy FJ , Gleeson LE , et al. Pyruvate kinase M2 regulates Hif‐1α activity and IL‐1β induction and is a critical determinant of the Warburg effect in LPS‐activated macrophages. Cell Metab. 2015;21(1):65–80. 25565206 10.1016/j.cmet.2014.12.005 PMC5198835

[46] Gatza E , Wahl DR , Opipari AW , Sundberg TB , Reddy P , Liu C , et al. Manipulating the bioenergetics of alloreactive T cells causes their selective apoptosis and arrests graft‐versus‐host disease. Sci Transl Med. 2011;3(67):67ra8. 10.1126/scitranslmed.3001975 PMC3364290 21270339

[47] Jha AK , Huang SCC , Sergushichev A , Lampropoulou V , Ivanova Y , Loginicheva E , et al. Network integration of parallel metabolic and transcriptional data reveals metabolic modules that regulate macrophage polarization. Immunity. 2015;42(3):419–430. 25786174 10.1016/j.immuni.2015.02.005

[48] Ali A , Davidson S , Fraenkel E , Gilmore I , Hankemeier T , Kirwan JA , et al. Single cell metabolism: current and future trends. Metabolomics. 2022;18:77. 36181583 10.1007/s11306-022-01934-3 PMC10063251

[49] Stephenson W , Donlin LT , Butler A , Rozo C , Bracken B , Rashidfarrokhi A , et al. Single‐cell RNA‐seq of rheumatoid arthritis synovial tissue using low‐cost microfluidic instrumentation. Nat Commun. 2018;9(1):791. 29476078 10.1038/s41467-017-02659-x PMC5824814

[50] Cochain C , Vafadarnejad E , Arampatzi P , Pelisek J , Winkels H , Ley K , et al. Single‐cell RNA‐seq reveals the transcriptional landscape and heterogeneity of aortic macrophages in murine atherosclerosis. Circ Res. 2018;122(12):1661–1674. 29545365 10.1161/CIRCRESAHA.117.312509

[51] Elmentaite R , Domínguez Conde C , Yang L , Teichmann SA . Single‐cell atlases: shared and tissue‐specific cell types across human organs. Nat Rev Genet. 2022;23:395–410. 35217821 10.1038/s41576-022-00449-w

[52] Winkels H , Ehinger E , Vassallo M , Buscher K , Dinh HQ , Kobiyama K , et al. Atlas of the immune cell repertoire in mouse atherosclerosis defined by single‐cell RNA‐sequencing and mass cytometry. Circ Res. 2018;122(12):1675–1688. 29545366 10.1161/CIRCRESAHA.117.312513 PMC5993603

[53] Vandereyken K , Sifrim A , Thienpont B , Voet T . Methods and applications for single‐cell and spatial multi‐omics. Nat Rev Genet. 2023;24:494–515. 36864178 10.1038/s41576-023-00580-2 PMC9979144

[54] Zielinski JM , Luke JJ , Guglietta S , Krieg C . High throughput multi‐omics approaches for clinical trial evaluation and drug discovery. Front Immunol. 2021;12:590742. 33868223 10.3389/fimmu.2021.590742 PMC8044891

[55] Sun C , Wang A , Zhou Y , Chen P , Wang X , Huang J , et al. Spatially resolved multi‐omics highlights cell‐specific metabolic remodeling and interactions in gastric cancer. Nat Commun. 2023;14(1):2692. 37164975 10.1038/s41467-023-38360-5 PMC10172194

[56] Su Y , Chen D , Yuan D , Lausted C , Choi J , Dai CL , et al. Multi‐omics resolves a sharp disease‐state shift between mild and moderate COVID‐19. Cell. 2020;183(6):1479–1495.e20. 33171100 10.1016/j.cell.2020.10.037 PMC7598382

[57] Schäfer KC , Dénes J , Albrecht K , Szaniszló T , Balog J , Skoumal R , et al. In vivo, in situ tissue analysis using rapid evaporative ionization mass spectrometry. Angew Chem Int Ed. 2009;48(44):8240–8242. 10.1002/anie.200902546 19746375

[58] Seubnooch P , Montani M , Tsouka S , Claude E , Rafiqi U , Perren A , et al. Characterisation of hepatic lipid signature distributed across the liver zonation using mass spectrometry imaging. JHEP Rep. 2023;5(6):100725. 37284141 10.1016/j.jhepr.2023.100725 PMC10240278

[59] Rosenberger FA , Thielert M , Strauss MT , Schweizer L , Ammar C , Mädler SC , et al. Spatial single‐cell mass spectrometry defines zonation of the hepatocyte proteome. Nat Methods. 2023;20(10):1530–1536. 37783884 10.1038/s41592-023-02007-6 PMC10555842

[60] Ma EH , Verway MJ , Johnson RM , Roy DG , Steadman M , Hayes S , et al. Metabolic profiling using stable isotope tracing reveals distinct patterns of glucose utilization by physiologically activated CD8+ T cells. Immunity. 2019;51(5):856–870.e5. 31747582 10.1016/j.immuni.2019.09.003

[61] Chometon TQ , Da Silva Siqueira M , Sant Anna JC , Almeida MR , Gandini M , De Almeida Nogueira ACM , et al. A protocol for rapid monocyte isolation and generation of singular human monocyte‐derived dendritic cells. PLoS One. 2020;15(4):e0231132. 32271804 10.1371/journal.pone.0231132 PMC7145147

[62] Kong BS , Lee C , Cho YM . Protocol for the assessment of human T cell activation by real‐time metabolic flux analysis. STAR Protoc. 2022;3(1):101084. 35072113 10.1016/j.xpro.2021.101084 PMC8761778

[63] Toda G , Yamauchi T , Kadowaki T , Ueki K . Preparation and culture of bone marrow‐derived macrophages from mice for functional analysis. STAR Protoc. 2021;2(1):100246. 33458708 10.1016/j.xpro.2020.100246 PMC7797923

[64] Sauter M , Sauter RJ , Nording H , Olbrich M , Emschermann F , Langer HF . Protocol to isolate and analyze mouse bone marrow derived dendritic cells (BMDC). STAR Protoc. 2022;3(3):101664. 36097382 10.1016/j.xpro.2022.101664 PMC9471451

[65] Chen KH , Boettiger AN , Moffitt JR , Wang S , Zhuang X . Spatially resolved, highly multiplexed RNA profiling in single cells. Science. 2015;348(6233):aaa6090. 25858977 10.1126/science.aaa6090 PMC4662681

[66] Fernández‐García J , Altea‐Manzano P , Pranzini E , Fendt SM . Stable isotopes for tracing mammalian‐cell metabolism in vivo. Trends Biochem Sci. 2020;45(3):185–201. 31955965 10.1016/j.tibs.2019.12.002

[67] Gubin MM , Esaulova E , Ward JP , Malkova ON , Runci D , Wong P , et al. High‐dimensional analysis delineates myeloid and lymphoid compartment remodeling during successful immune‐checkpoint cancer therapy. Cell. 2018;175(4):1014–1030.e19. 30343900 10.1016/j.cell.2018.09.030 PMC6501221

[68] Danzi F , Pacchiana R , Mafficini A , Scupoli MT , Scarpa A , Donadelli M , et al. To metabolomics and beyond: a technological portfolio to investigate cancer metabolism. Signal Transduct Target Ther. 2023;8:137. 36949046 10.1038/s41392-023-01380-0 PMC10033890

[69] Abouleila Y , Onidani K , Ali A , Shoji H , Kawai T , Lim CT , et al. Live single cell mass spectrometry reveals cancer‐specific metabolic profiles of circulating tumor cells. Cancer Sci. 2019;110(2):697–706. 30549153 10.1111/cas.13915 PMC6361580

[70] Bensen RC , Standke SJ , Colby DH , Kothapalli NR , Le‐Mcclain AT , Patten MA , et al. Single cell mass spectrometry quantification of anticancer drugs: proof of concept in cancer patients. ACS Pharmacol Transl Sci. 2021;4(1):96–100. 33615163 10.1021/acsptsci.0c00156 PMC7887743

[71] Hu R , Li Y , Yang Y , Liu M . Mass spectrometry‐based strategies for single‐cell metabolomics. Mass Spectrom Rev. 2023;42:67–94. 34028064 10.1002/mas.21704

[72] Llufrio EM , Wang L , Naser FJ , Patti GJ . Sorting cells alters their redox state and cellular metabolome. Redox Biol. 2018;16:381–387. 29627745 10.1016/j.redox.2018.03.004 PMC5952879

[73] Argüello RJ , Combes AJ , Char R , Gigan JP , Baaziz AI , Bousiquot E , et al. SCENITH: a flow cytometry‐based method to functionally profile energy metabolism with single‐cell resolution. Cell Metab. 2020;32(6):1063–1075.e7. 33264598 10.1016/j.cmet.2020.11.007 PMC8407169

[74] Ahl PJ , Hopkins RA , Xiang WW , Au B , Kaliaperumal N , Fairhurst AM , et al. Met‐Flow, a strategy for single‐cell metabolic analysis highlights dynamic changes in immune subpopulations. Commun Biol. 2020;3(1):305. 32533056 10.1038/s42003-020-1027-9 PMC7292829

[75] Adamik J , Munson PV , Hartmann FJ , Combes AJ , Pierre P , Krummel MF , et al. Distinct metabolic states guide maturation of inflammatory and tolerogenic dendritic cells. Nat Commun. 2022;13(1):5184. 36056019 10.1038/s41467-022-32849-1 PMC9440236

[76] Park LM , Lannigan J , Jaimes MC . OMIP‐069: forty‐color full spectrum flow cytometry panel for deep immunophenotyping of major cell subsets in human peripheral blood. Cytometry A. 2020;97(10):1044–1051. 32830910 10.1002/cyto.a.24213 PMC8132182

[77] Stoeckius M , Hafemeister C , Stephenson W , Houck‐Loomis B , Chattopadhyay PK , Swerdlow H , et al. Simultaneous epitope and transcriptome measurement in single cells. Nat Methods. 2017;14(9):865–868. 28759029 10.1038/nmeth.4380 PMC5669064

[78] Su Y , Yuan D , Chen DG , Ng RH , Wang K , Choi J , et al. Multiple early factors anticipate post‐acute COVID‐19 sequelae. Cell. 2022;185(5):881–895.e20. 35216672 10.1016/j.cell.2022.01.014 PMC8786632

[79] Leader AM , Grout JA , Maier BB , Nabet BY , Park MD , Tabachnikova A , et al. Single‐cell analysis of human non‐small cell lung cancer lesions refines tumor classification and patient stratification. Cancer Cell. 2021;39(12):1594–1609.e12. 34767762 10.1016/j.ccell.2021.10.009 PMC8728963

[80] Wu SZ , Al‐Eryani G , Roden DL , Junankar S , Harvey K , Andersson A , et al. A single‐cell and spatially resolved atlas of human breast cancers. Nat Genet. 2021;53(9):1334–1347. 34493872 10.1038/s41588-021-00911-1 PMC9044823

[81] Frangieh CJ , Melms JC , Thakore PI , Geiger‐Schuller KR , Ho P , Luoma AM , et al. Multimodal pooled perturb‐CITE‐seq screens in patient models define mechanisms of cancer immune evasion. Nat Genet. 2021;53(3):332–341. 33649592 10.1038/s41588-021-00779-1 PMC8376399

[82] Hartmann FJ , Mrdjen D , McCaffrey E , Glass DR , Greenwald NF , Bharadwaj A , et al. Single‐cell metabolic profiling of human cytotoxic T cells. Nat Biotechnol. 2021;39(2):186–197. 32868913 10.1038/s41587-020-0651-8 PMC7878201

[83] Levine LS , Hiam‐Galvez KJ , Marquez DM , Tenvooren I , Madden MZ , Contreras DC , et al. Single‐cell analysis by mass cytometry reveals metabolic states of early‐activated CD8+ T cells during the primary immune response. Immunity. 2021;54(4):829–844.e5. 33705706 10.1016/j.immuni.2021.02.018 PMC8046726

[84] Alexandrov T . Spatial metabolomics and imaging mass spectrometry in the age of artificial intelligence. Annual Rev Biomed Data Sci. 2020;3:61–87. 34056560 10.1146/annurev-biodatasci-011420-031537 PMC7610844

[85] Vaysse PM , Heeren RMA , Porta T , Balluff B . Mass spectrometry imaging for clinical research‐latest developments, applications, and current limitations. Analyst. 2017;142:2690–2712. 28642940 10.1039/c7an00565b

[86] Gilmore IS , Heiles S , Pieterse CL . Metabolic imaging at the single‐cell scale: recent advances in mass spectrometry imaging. Annu Rev Anal Chem. 2019;12(1):201–224. 10.1146/annurev-anchem-061318-115516 30848927

[87] Greco F , Quercioli L , Pucci A , Rocchiccioli S , Ferrari M , Recchia FA , et al. Mass spectrometry imaging as a tool to investigate region specific lipid alterations in symptomatic human carotid atherosclerotic plaques. Metabolites. 2021;11(4):250. 33919525 10.3390/metabo11040250 PMC8073208

[88] Rappez L , Stadler M , Triana S , Gathungu RM , Ovchinnikova K , Phapale P , et al. SpaceM reveals metabolic states of single cells. Nat Methods. 2021;18(7):799–805. 34226721 10.1038/s41592-021-01198-0 PMC7611214

[89] Holzlechner M , Strasser K , Zareva E , Steinhäuser L , Birnleitner H , Beer A , et al. In situ characterization of tissue‐resident immune cells by MALDI mass spectrometry imaging. J Proteome Res. 2017;16(1):65–76. 27755872 10.1021/acs.jproteome.6b00610

[90] Cuypers E , Claes BSR , Biemans R , Lieuwes NG , Glunde K , Dubois L , et al. On the spot’ digital pathology of breast cancer based on single‐cell mass spectrometry imaging. Anal Chem. 2022;94(16):6180–6190. 35413180 10.1021/acs.analchem.1c05238 PMC9047448

[91] Goossens P , Lu C , Cao J , Gijbels MJ , Karel JMH , Wijnands E , et al. Integrating multiplex immunofluorescent and mass spectrometry imaging to map myeloid heterogeneity in its metabolic and cellular context. Cell Metab. 2022;34(8):1214–1225.e6. 35858629 10.1016/j.cmet.2022.06.012

[92] Rabold K , Aschenbrenner A , Thiele C , Boahen CK , Schiltmans A , Smit JWA , et al. Enhanced lipid biosynthesis in human tumor‐induced macrophages contributes to their protumoral characteristics. J Immunother Cancer. 2020;8(2):e000638. 32943450 10.1136/jitc-2020-000638 PMC7500191

[93] Trapecar M , Communal C , Velazquez J , Maass CA , Huang YJ , Schneider K , et al. Gut‐liver physiomimetics reveal paradoxical modulation of IBD‐related inflammation by short‐chain fatty acids. Cell Syst. 2020;10(3):223–239.e9. 32191873 10.1016/j.cels.2020.02.008 PMC8143761

[94] Bein A , Kim S , Goyal G , Cao W , Fadel C , Naziripour A , et al. Enteric coronavirus infection and treatment modeled with an immunocompetent human intestine‐on‐A‐chip. Front Pharmacol. 2021;12:718484. 34759819 10.3389/fphar.2021.718484 PMC8573067

[95] Medlock GL , Carey MA , McDuffie DG , Mundy MB , Giallourou N , Swann JR , et al. Inferring metabolic mechanisms of interaction within a defined gut microbiota. Cell Syst. 2018;7(3):245–257.e7. 30195437 10.1016/j.cels.2018.08.003 PMC6166237

[96] Thiam F , Al Yazeedi S , Feng K , Phogat S , Demirsoy E , Brussow J , et al. Understanding fibroblast‐immune cell interactions via co‐culture models and their role in asthma pathogenesis. Front Immunol. 2023;14:1128023. 36911735 10.3389/fimmu.2023.1128023 PMC9996007

[97] Lagies S , Schlimpert M , Neumann S , Wäldin A , Kammerer B , Borner C , et al. Cells grown in three‐dimensional spheroids mirror in vivo metabolic response of epithelial cells. Commun Biol. 2020;3(1):246. 32427948 10.1038/s42003-020-0973-6 PMC7237469

[98] de Kivit S , Mensink M , Hoekstra AT , Berlin I , Derks RJE , Both D , et al. Stable human regulatory T cells switch to glycolysis following TNF receptor 2 costimulation. Nat Metab. 2020;2(10):1046–1061. 32958937 10.1038/s42255-020-00271-w

[99] Siska PJ , Jiao J , Matos C , Singer K , Dettmer K , Oefner PJ , et al. Kynurenine induces T cell fat catabolism and has limited suppressive effects in vivo. EBioMedicine. 2021;74:103734. 34875457 10.1016/j.ebiom.2021.103734 PMC8652007

[100] Mensink M , Tran TNM , Zaal EA , Schrama E , Berkers CR , Borst J , et al. TNFR2 costimulation differentially impacts regulatory and conventional CD4+ T‐cell metabolism. Front Immunol. 2022;13:881166. 35844585 10.3389/fimmu.2022.881166 PMC9282886

[101] Matamala Montoya M , van Slobbe GJJ , Chang JC , Zaal EA , Berkers CR . Metabolic changes underlying drug resistance in the multiple myeloma tumor microenvironment. Front Oncol. 2023;13:1155621. 37091139 10.3389/fonc.2023.1155621 PMC10117897

[102] Chang CH , Curtis JD , Maggi LB , Faubert B , Villarino AV , O'Sullivan D , et al. Posttranscriptional control of T cell effector function by aerobic glycolysis. Cell. 2013;153(6):1239–1251. 23746840 10.1016/j.cell.2013.05.016 PMC3804311

[103] Qiu J , Villa M , Sanin DE , Buck MD , O'Sullivan D , Ching R , et al. Acetate promotes T cell effector function during glucose restriction. Cell Rep. 2019;27(7):2063–2074.e5. 31091446 10.1016/j.celrep.2019.04.022 PMC6544383

[104] Wagner A , Wang C , Fessler J , DeTomaso D , Avila‐Pacheco J , Kaminski J , et al. Metabolic modeling of single Th17 cells reveals regulators of autoimmunity. Cell. 2021;184(16):4168–4185.e21. 34216539 10.1016/j.cell.2021.05.045 PMC8621950

[105] Puleston DJ , Baixauli F , Sanin DE , Edwards‐Hicks J , Villa M , Kabat AM , et al. Polyamine metabolism is a central determinant of helper T cell lineage fidelity. Cell. 2021;184(16):4186–4202.e20. 34216540 10.1016/j.cell.2021.06.007 PMC8358979

[106] Curtis M , Kenny HA , Ashcroft B , Mukherjee A , Johnson A , Zhang Y , et al. Fibroblasts mobilize tumor cell glycogen to promote proliferation and metastasis. Cell Metab. 2019;29(1):141–155.e9. 30174305 10.1016/j.cmet.2018.08.007 PMC6326875

[107] Wang G , Heijs B , Kostidis S , Mahfouz A , Rietjens RGJ , Bijkerk R , et al. Analyzing cell‐type‐specific dynamics of metabolism in kidney repair. Nat Metab. 2022;4(9):1109–1118. 36008550 10.1038/s42255-022-00615-8 PMC9499864

[108] Djekic D , Nicoll R , Novo M , Henein M . Metabolomics in atherosclerosis. IJC Metab Endocr. 2015;8:26–30.

[109] Yang K , Zhang F , Han P , Wang ZZ , Deng K , Zhang YY , et al. Metabolomics approach for predicting response to neoadjuvant chemotherapy for colorectal cancer. Metabolomics. 2018;14(9):110. 30830371 10.1007/s11306-018-1406-0

[110] Odom JD , Sutton VR . Metabolomics in clinical practice: improving diagnosis and informing management. Clin Chem. 2021;67:1606–1617. 34633032 10.1093/clinchem/hvab184

[111] Schmidt DR , Patel R , Kirsch DG , Lewis CA , Vander Heiden MG , Locasale JW . Metabolomics in cancer research and emerging applications in clinical oncology. CA Cancer J Clin. 2021;71(4):333–358. 33982817 10.3322/caac.21670 PMC8298088

[112] Xiao Y , Ma D , Yang YS , Yang F , Ding JH , Gong Y , et al. Comprehensive metabolomics expands precision medicine for triple‐negative breast cancer. Cell Res. 2022;32(5):477–490. 35105939 10.1038/s41422-022-00614-0 PMC9061756

[113] Roberts LD , Souza AL , Gerszten RE , Clish CB . Targeted metabolomics. Curr Protoc Mol Biol. 2012;98(1):1–24. 10.1002/0471142727.mb3002s98 PMC3334318 22470063

[114] Coene KLM , Kluijtmans LAJ , van der Heeft E , Engelke UFH , de Boer S , Hoegen B , et al. Next‐generation metabolic screening: targeted and untargeted metabolomics for the diagnosis of inborn errors of metabolism in individual patients. J Inherit Metab Dis. 2018;41(3):337–353. 29453510 10.1007/s10545-017-0131-6 PMC5959972

[115] Hertzog A , Selvanathan A , Devanapalli B , Ho G , Bhattacharya K , Tolun AA . A narrative review of metabolomics in the era of “‐omics”: integration into clinical practice for inborn errors of metabolism. Transl Pediatr. 2022;11(10):1704–1716. 36345452 10.21037/tp-22-105 PMC9636448

[116] Karagiannis F , Peukert K , Surace L , Michla M , Nikolka F , Fox M , et al. Impaired ketogenesis ties metabolism to T cell dysfunction in COVID‐19. Nature. 2022;609(7928):801–807. 35901960 10.1038/s41586-022-05128-8 PMC9428867

[117] Roach PJ , Laskin J , Laskin A . Nanospray desorption electrospray ionization: an ambient method for liquid‐extraction surface sampling in mass spectrometry. Analyst. 2010;135(9):2233–2236. 20593081 10.1039/c0an00312c

[118] Samarah LZ , Khattar R , Tran TH , Stopka SA , Brantner CA , Parlanti P , et al. Single‐cell metabolic profiling: metabolite formulas from isotopic fine structures in heterogeneous plant cell populations. Anal Chem. 2020;92(10):7289–7298. 32314907 10.1021/acs.analchem.0c00936

[119] Taylor MJ , Lukowski JK , Anderton CR . Spatially resolved mass spectrometry at the single cell: recent innovations in proteomics and metabolomics. J Am Soc Mass Spectrom. 2021;32(4):872–894. 33656885 10.1021/jasms.0c00439 PMC8033567

[120] Ruiz‐Rodado V , Lita A , Larion M . Advances in measuring cancer cell metabolism with subcellular resolution. Nat Methods. 2022;19:1048–1063. 36008629 10.1038/s41592-022-01572-6

[121] Llufrio EM , Cho K , Patti GJ . Systems‐level analysis of isotopic labeling in untargeted metabolomic data by X13CMS. Nat Protoc. 2019;14(7):1970–1990. 31168088 10.1038/s41596-019-0167-1 PMC7323898

[122] Puchalska P , Huang X , Martin SE , Han X , Patti GJ , Crawford PA . Isotope tracing untargeted metabolomics reveals macrophage polarization‐state‐specific metabolic coordination across intracellular compartments. iScience. 2018;9:298–313. 30448730 10.1016/j.isci.2018.10.029 PMC6240706

[123] Wang M , Carver JJ , Phelan V , Sanchez LM , Garg N , Peng Y , et al. Sharing and community curation of mass spectrometry data with global natural products social molecular networking. Nat Biotechnol. 2016;34:828–837. 27504778 10.1038/nbt.3597 PMC5321674

[124] Chang TC , Mikheev AM , Huynh W , Monnat RJ , Rostomily RC , Folch A . Parallel microfluidic chemosensitivity testing on individual slice cultures. Lab Chip. 2014;14(23):4540–4551. 25275698 10.1039/c4lc00642a PMC4217250

[125] Chen X , Peng Z , Yang Z . Metabolomics studies of cell‐cell interactions using single cell mass spectrometry combined with fluorescence microscopy. Chem Sci. 2022;13(22):6687–6695. 35756524 10.1039/d2sc02298b PMC9172575

